# Efficacy of methylprednisolone on pain, trismus and quality of life following surgical removal of mandibular third molars: a double-blind, split-mouth, randomised controlled trial

**DOI:** 10.4317/medoral.24094

**Published:** 2020-07-23

**Authors:** Marie Kjærgaard Larsen, Thomas Kofod, Kirsten Duch, Thomas Starch-Jensen

**Affiliations:** 1 Department of Oral and Maxillofacial Surgery, Aalborg University Hospital, Denmark; 2 Department of Oral and Maxillofacial Surgery, Rigshospitalet, Copenhagen University Hospital, Denmark; 3 Unit of Clinical Biostatistics, Aalborg University Hospital, Aalborg, Denmark

## Abstract

**Background:**

The objective of the present study was to compare the efficacy of different doses of methylprednisolone on postoperative sequelae and quality of life (QoL) following surgical removal of mandibular third molars (SRM3).

**Material and Methods:**

Fifty-two patients (16 men and 36 women, mean age 25.9 years, range: 18-39) with bilateral impacted mandibular third molars were randomly allocated into intraoperative muscular injection of either 20mg, 30mg, 40mg methylprednisolone or saline injection. Baseline measurements were obtained preoperatively and compared with assessment after one day, three days, seven days and one month. Pain and trismus were estimated by visual analog scale score and interincisal mouth opening, respectively. Subjective assessment of QoL included Oral Health Impact Profile (OHIP-14). Descriptive and generalized estimating equation analyses were made and expressed as mean values with a 95% confidence interval.

**Results:**

Methylprednisolone revealed no significant differences in pain, trismus and QoL compared with placebo. Higher prevalence of postoperative pain and worsening in QoL were observed with increased age (*P*=0.00). Smoking and increased time of surgery decreased mouth opening in the early healing phase (*P*=0.00).

**Conclusions:**

The present study revealed no significant improvement of methylprednisolone on postoperative sequelae and QoL following SRM3 compared with placebo.

** Key words:**Corticosteroids, dentistry, mandible, methylprednisolone, pain, third molar, trismus.

## Introduction

Swelling, pain and trismus are common sequelae following surgical removal of mandibular third molars (SRM3) (1). Synthetic corticosteroids display anti-inflammatory properties including reduction in vascular dilatation, liquid transudation and edema formation (2), and therefore widely used to control or diminish the inflammatory response associated with SRM3 (3,4). Systematic reviews and meta-analyses have evaluated efficacy of corticosteroids to diminish postoperative sequelae following SRM3 with conflicting conclusions (3,5,6). Thus, no evidence-based recommendations for optimal administration route, dose and therapy duration of corticosteroids have been provided (3).

Comparable efficacy of corticosteroids on postoperative sequelae following SRM3 with different administration route have previously been reported (7-10), whereas diminished swelling, pain and trismus have been described with higher doses of corticosteroids (11,12). These results are in contrast to a systematic review concluding that higher doses of corticosteroids do not necessarily cause a proportionally decrease in swelling, pain and trismus (3). However, studies comparing different doses of corticosteroids following SRM3 are limited and no evidence-based recommendation for optimal dose has previously been provided (3,13). Therefore, the objective of this double-blind randomised controlled trial was to assess the efficacy of different doses of methylprednisolone on pain, trismus and quality of life (QoL) compared with placebo following SRM3.

## Material and Methods

- Study design

A double-blinded, split-mouth, randomised controlled trial was carried out at the Department of Oral and Maxillofacial Surgery, Aalborg University Hospital, Denmark between March 2018 and January 2019. The study protocol was approved by the Danish Health and Medicines Authority, Research Ethics Committee and the Danish Data Protection Agency. Approval no.: 20170016. The study was performed in accordance with Good Clinical Practice (GCP), Declaration of Helsinki II and Consolidated Standards of Reporting Trials (CONSORT) statement. Oral and written information were provided and written informed consent was obtained prior to enrolment. Patients were recruited by personal contacts, public invitation by Facebook or scheduled for SRM3 prior to orthognathic surgery. Participation was voluntary and patients could at any given time withdraw.

- Power calculation

The sample size was determined using an expected difference of 20mm in visual analog scale (VAS) score between placebo and treatment on the first postoperative day and a standard deviation of two. The VAS score difference of 20mm in pain assessment after SRM3 was selected based on previous studies evaluating identical parameters (14,15). Sample size was calculated using Clincalc.com (http://clincalc.com/stats/samplesize.aspx, assessed 9th March 2017). Analysis revealed that 16 patients per group would be necessary to provide statistical power of 0.80 with an alpha value of 0.05. Sample size was increased to 26 SRM3 to compensate for possible dropouts and covariates.

- Study population

The inclusion criteria were:

1. Bilateral symmetrical impacted mandibular third molars

2. Indication for removal of mandibular third molars

3. Age between 18 and 40 years

The exclusion criteria were:

1. Infections and inflammatory symptoms in the oral cavity at the time of surgery

2. ASA score 3 or above

3. Previous maxillofacial trauma

4. Craniofacial clefts or syndromes

5. Known allergy to methylprednisolone and other inactive ingredients

6. Systemic bone disease (i.e. arthritis) or diabetes mellitus

7. Active acne vulgaris, viral, and fungal infections

8. Psychological disease

9. Pregnancy and breastfeeding

10. Systemic medications

11. Failure to follow-up

Panoramic radiograph was used to categorize the position of M3 according to Pell and Gregory system and Winters as well as the surgical difficulty level of the M3 classification.

- Randomization

The pharmacy at Aalborg University Hospital, Denmark distributes Methylprednisolone in 40mg solution for injection. The following four groups were therefore investigated: I: placebo (isotonic saline solution), II: 20mg methylprednisolone, III: 30mg methylprednisolone and IV: 40mg methylprednisolone. A computer-aided randomization scheme was fabricated by the pharmacy including randomization numbers and allocation group for each M3. A trained assistant nurse prepared syringes containing the different mixture of isotonic saline solution and doses of methylprednisolone. The concentration of methylprednisolone from the pharmacy was 40mg/mL. All syringes contained 1.05mL of clear liquid. Therefore, the syringes containing 20mg methylprednisolone contained 0.5mL of 40mg/mL methylprednisolone, the syringes containing 30mg methylprednisolone contained 0.75mL of 40mg/mL methylprednisolone, and the syringes containing 40mg methylprednisolone contained 1mL of 40mg/mL methylprednisolone. Patients, surgeon, dental assistant or assessor were not informed about allocation group or solution of syringes.

- Surgical procedure

SRM3 was performed by an experienced surgeon (MKL) using a standard technique. Patients underwent SRM3 (left or right) at first visit, while the other M3 was removed after 58.8 days (range; 8-157 days).

All patients received prophylactic analgesic, 400mg ibuprofen and 1,000mg paracetamol, one hour before surgery. Inferior alveolar nerve and lingual nerve were anaesthetized with 5.37mL (range: 5-10mL) 20mg/mL mepivacaine hydrochloride and 5µg/mL adrenaline. Injection preceded by aspiration of placebo or methylprednisolone was performed immediately after application of local anaesthesia in the ipsilateral masseter muscle. The ala-tragal line, the posterior border line of masseter and the mandible border line were used as landmarks for injection of placebo or methylprednisolone. An incision from the anterior border of the ascending ramus of the mandible to the distal part of the lower first molar was performed. The mucosal flap was elevated and bone around the M3 was removed with a round burr under irrigation with 0.9% saline solution. If necessary, the M3 was sectioned with a fissure bur before the tooth was elevated. Extraction socket and surround bone was irrigated with 0.9% saline solution before suturing.

All patients received postoperative instructions including mouth rinse with 0.12% chlorhexidine three times a day, 400mg of ibuprofen three times a day and 1,000mg paracetamol four times a day.

- Data collection

Data was collected by the same assessor (MKL). Baseline measurements were obtained preoperatively (T0) and compared with postoperative assessment after one day (T1), three days (T2), seven days (T3) and one month (T4), respectively.

Pain was evaluated using VAS score obtained preoperatively (T0) and compared with the postoperative score at T1, T2, T3 and T4, respectively. Patients were instructed in the use of a 100-mm VAS scale with 0 indicating no pain and 100 indicating worst imaginable pain. Patients marked on a line the point that they felt represented their level pain. VAS score was measured to the nearest mm using a ruler from left to the point marked by the patient.

Trismus was measured as maximum distance (mm) between upper and lower incisal edges. Baseline measurements were obtained preoperatively (T0) and compared with postoperative measurements obtained at T2, T3 and T4, respectively.

QoL was evaluated by oral health impact profile-14 (OHIP-14). Instructions for completing OHIP-14 were explained, before patients completed OHIP-14 by themselves, to prevent being influenced by the surgeon or nurses opinions and wills. OHIP-14 was filled-out preoperatively (T0) and compared with OHIP-14 at T3 and T4. Patients were specifically instructed to complete the OHIP-14 questionnaire separately for the corresponding M3, if there was overlap in the postoperative period with regard to completing OHIP-14 questionnaire after SRM3 in the right or left side, respectively. The response format of OHIP-14 was as follows: All the time=4; Very often=3; Fairly often=2; Sometimes=1; Never=0. The OHIP-14 score was calculated as a sum of all 14 questions ranging from 0 to 56, with higher scores indicating poorer oral health related QOL.

Postoperative complications were registered at T2, T3 and T4.

- Statistical analysis

Excel (version 2013, Microsoft, Redmond, Washington) and R (version 3.6.1, Missouri, USA) was used for data management and statistical analysis. Difference in VAS, trismus and OHIP-14 were analyzed with a generalized estimating equation analysis, GEE analysis. Results were adjusted for age, sex, smoking and time of surgery. *P* values of less than 0.05 were considered significant.

Descriptive analysis of secondary categorical variables including smoking habits, anatomical position of M3 and infection rate was analysed using Fishers exact test.

## Results

- Study population

Fifty-two patients (16 men and 36 female) aged between 18 and 39 years (25.9±6.0 years) were included for statistical analysis. One patient did not participate in the follow-up phase and was excluded from the analysis. There were no statistical significant differences between the study groups with regard to smoking (*P*=0.836), anatomical position or surgical difficulty level (*P*=0.660) and time of surgery (*P*=0.330) (Table 1). Mean surgical time was 9.42 minutes (±5.18). Patients with smoking habits included cigarette smokers only.

Postoperative instructions were followed by all patients. Two patients (1.9%) experienced bleeding within the first hours after SRM3. None of the included patients needed additional prescriptions of analgesics. Swelling, discomfort, tenderness and halitosis were reported sporadically. No serious postoperative complications or neurosensory disturbances were observed.

- Pain

**Figure 1:**
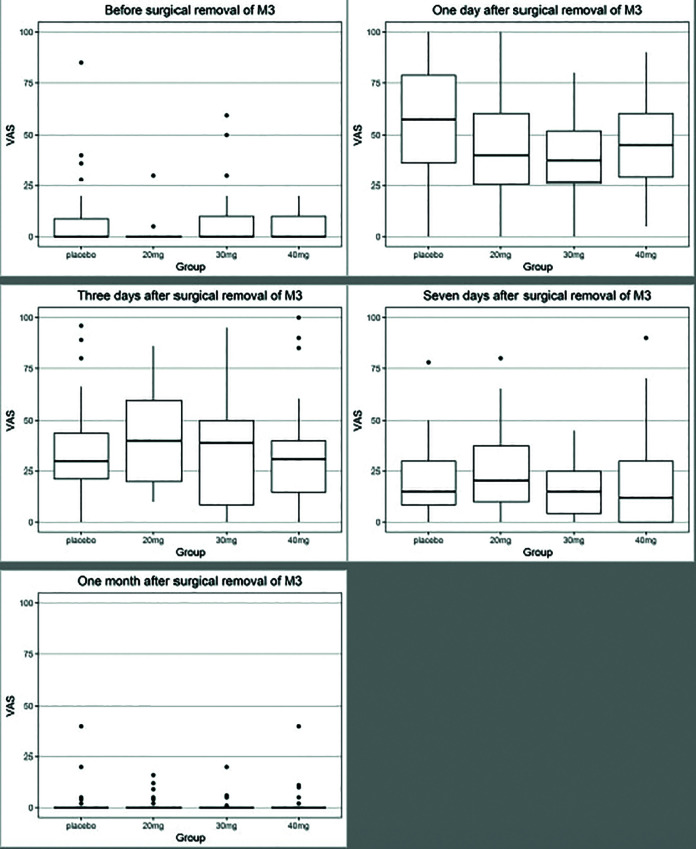
Boxplot of VAS.

Mean VAS score was 6.27±13.98 (T0), 45.58±24.41 (T1), 36.88±25.26 (T2), 21.00±20.35 (T3), and 2.24±6.59 (T4). There were no significant differences in VAS score between different doses of methylprednisolone compared with placebo at any time point (Table 2). Patients receiving 30mg methylprednisolone reported a tendency to diminished pain compared with placebo or 20mg and 40mg of methylprednisolone at any time point (Table 2 and Fig. 1).

**Table 1:**
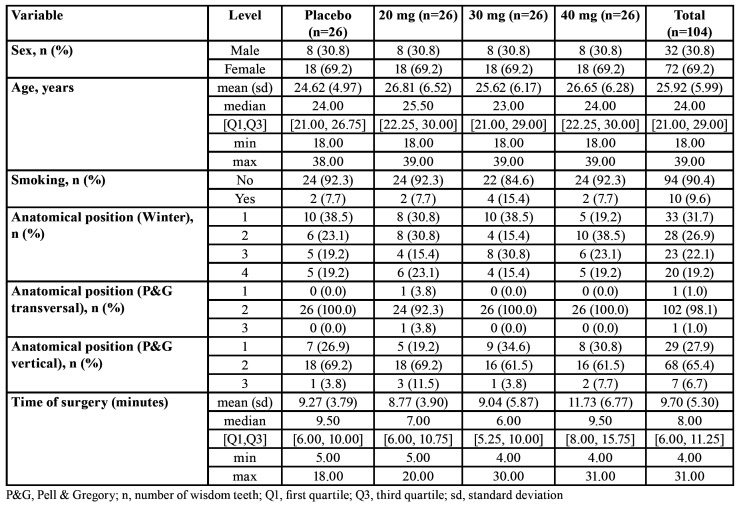
Baseline characteristics, anatomical position of mandibular wisdom teeth and time of surgery in the four groups and total.

**Table 2:**
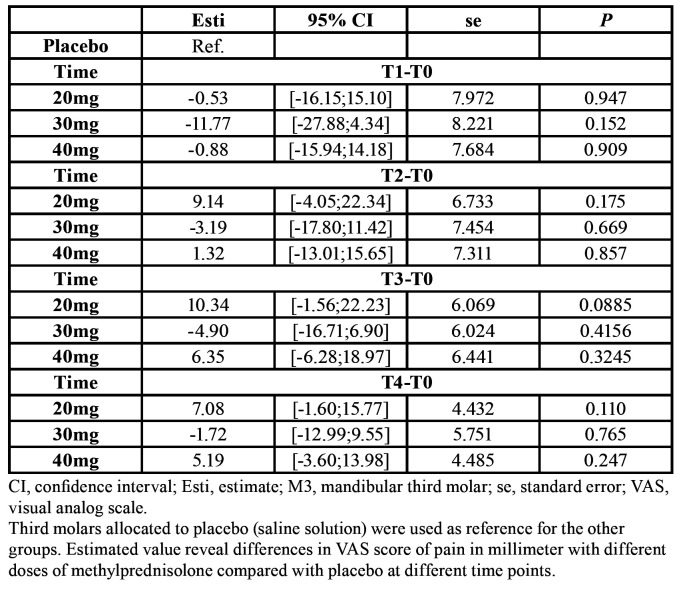
Pain assessed by VAS before removal of M3 (T0) compared with one day (T1), three days (T2), seven days (T3) and one month (T4)

Patients with increased age revealed a significant higher VAS score after seven days and one month (*P*=0.01). The VAS score increased continuously by 1.3mm after seven days and 0.53mm after one month, when patient’s age increased with one year. Males presented with a significant higher VAS score of 5.83mm compared with females after one month (*P*=0.02).

- Trismus

Mean mouth opening was 50.76±5.85 (T0), 32.49±9.55 (T1), 38.07±9.44 (T2), 21.00±20.35 (T3), and 50.43±6.82 (T4). There were no significant differences in trismus between different doses of methylprednisolone compared with placebo at any time point (Table 3).

A significant difference was assessed in mouth opening between smokers and non-smokers after three days and seven days (*P*=0.00). Mouth opening was reduced in smokers by 6.29mm (T3) and 7.76mm (T4) compared with non-smokers. A significant difference in mouth opening was seen with an increased time of surgery after three days (*P*=0.04). Mouth opening continuously decreased by 0.37mm, when time of surgery increased by one minute.

- Quality of life

Mean OHIP-14 score was 7.89±7.94 (T0), 16.06±12.13 (T3), and 5.77±8.22 (T4). There were no significant differences in QoL between different doses of methylprednisolone compared with placebo at any time point. In each group, there were significant differences in the sum of OHIP-14 score between T0 and T3 (*P*=0.00). Though, no significant differences were seen in QoL between placebo and different doses of methylprednisolone (Table 4).

A significant difference in OHIP-14 score was observed with an increased age, after seven days (T3) (*P*=0.04). The OHIP-14 score increased continuously with 0.56 after seven days, when patient’s age increased with one year.

**Table 3:**
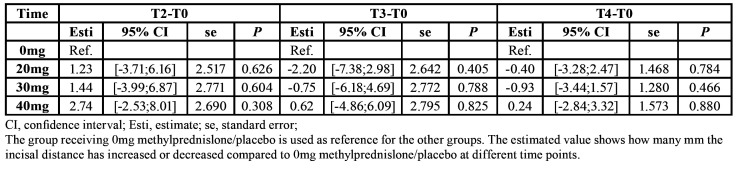
Trismus results (difference between before surgery and T2, T3 and T4).

**Table 4:**
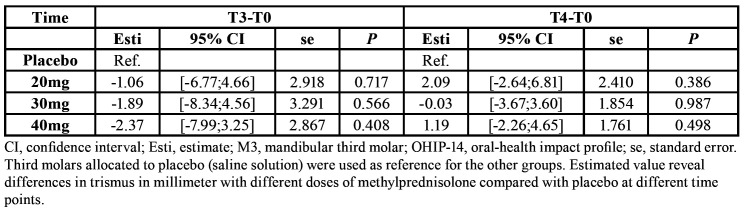
OHIP-14 score before removal of M3 (T0) compared with seven days (T3) and one month (T4).

## Discussion

Comparison of different doses of corticosteroids on postoperative sequelae and QoL following SRM3 has previously been assessed in few studies (16,17). Submucosal and peroral administration of prednisolone were associated with improved recovery and less worsening in QoL compared with placebo (16). The efficacy of different doses of corticosteroids compared with placebo is conflicting and conclusions from the present study seems to be in accordance with previous studies, indicating no significant improvement in postoperative sequelae and QoL with a single intraoperative intramuscular injection of corticosteroids compared with placebo following SRM3 (16,17). Moreover, higher doses of corticosteroids seem not to cause a proportionally improvement in postoperative sequelae and QoL.

Pain has been reported as the worst postoperative sequelae following SRM3 (18). Thus, intensity of pain during the first postoperative day was chosen as the primary outcome measure of the present study. VAS is a validated, subjective measure for assessment and analysis of postoperative pain (18). The present study demonstrated no significantly difference in VAS score of pain following SRM3 with different doses of methylprednisolone compared with placebo on the first postoperative day, which is in agreement with previous studies (11,12). Moreover, VAS score of pain significantly increased with higher age, which is also in accordance with previous studies (19,20). Perception of postoperative pain correlated with gender have previous been assessed revealing that females seems to be at higher risk of postoperative pain (21,22). In the present study, males disclosed a significantly higher VAS score of pain compared with females after one month. Increased time of surgery has previously been reported to increase risk of pain(18). However, this could not be substantiated in the present study. In conclusion, a single dose of methylprednisolone seems not to diminish VAS score of pain compared with placebo following SRM3.

Trismus following SRM3 is common (1). The present study demonstrated no significantly difference in trismus following SRM3 with different doses of methylprednisolone compared with placebo, which is in agreement with previous studies (23,24). However, a recent published study revealed a significantly improvement in mouth opening following SRM3 with submucosal administration of 40mg methylprednisolone compared with placebo (12). Moreover, peroral administration of 8mg dexamethasone (approximate: 40mg methylprednisolone) has demonstrated a significantly improvement in postoperative mouth opening compared with 4mg dexamethasone (approximate: 20mg methylprednisolone) (11). Trismus may therefore been diminish with the use of higher doses of corticosteroids, which should be investigated in further studies. In the present study, smokers demonstrated significantly diminish mouth opening compared with non-smokers after three days and seven days, which is in accordance with previous studies (25,26). However, degree of smoking was not categorised and ranged from rarely to more than 20 cigarettes daily, which affect the reliability of the present result. Moreover, no significantly difference in mouth opening has previously been reported among smokers compared with non-smokers following SRM3 (27). In the present study, increased time of surgery demonstrated a significantly reduction in postoperative mouth opening, which is in accordance with a previous study (26).

Deterioration in QoL is common following SRM3 (28). The present study revealed no significant differences in QoL as evaluated by OHIP-14 following SRM3 with different doses of methylprednisolone compared with placebo. A recent randomised controlled trial has demonstrated improved QoL with submucosal and peroral administration of 40mg prednisolone (approximate: 32mg methylprednisolone) compared with placebo (16). Moreover, submucosal and intramuscular administration of 4mg dexamethasone (approximate: 20mg methylprednisolone) has revealed a positive effect on QoL compared with placebo (16). These results are in contrast to the present study and illustrates that QoL might be improved with a lower dose of corticosteroids. In the present study, OHIP-14 score was significantly increased with higher age after seven days, indicating deterioration in QoL with increasing age. These results is in accordance with previous studies (28,29). Females are reported to have a higher risk of poor recovery and worsening in QoL after SRM3 compared with males (26). However, gender seems not to influence QoL in the present study. Increased time of surgery is reported to influence QoL (28), which is in contrast to results of the present study. In conclusion, methylprednisolone seems not to diminish QoL compared with placebo following SRM3. Though, increasing age seems to be associated with deterioration in QoL.

Adverse effects following use of corticosteroids depends on doses and duration (30). Adverse effects to a single dose of corticosteroids after SRM3 have never previously been reported (7,13), which is in accordance with the present study.

The present study has several limitations including small sample size, dissimilar gender distribution, smokers as well as non-smokers included and no systematically registration of the used analgesics. The surgical procedures and data assessment were conducted by the same investigator, though decoding of allocation groups were revealed after data processing. Moreover, patient’s perception of recovery and oral health-related quality of life is influenced by socioeconomic status, educational background and level of daily physical, which were not assessed in the present study. Consequently, conclusions drawn from the results of this study should be interpreted with caution and further randomised controlled trials including larger patient samples, homogenous study groups and comparison of higher doses of corticosteroids are needed before definite conclusions can be provided about the influence of corticosteroids on postoperative sequelae and QoL following SRM3.
